# Rare Pathogenic Variants in Pooled Whole-Exome Sequencing Data Suggest Hyperammonemia as a Possible Cause of Dementia Not Classified as Alzheimer’s Disease or Frontotemporal Dementia

**DOI:** 10.3390/genes15060753

**Published:** 2024-06-07

**Authors:** Sena Karachanak-Yankova, Dimitar Serbezov, Georgi Antov, Mikaela Stancheva, Marta Mihaylova, Savina Hadjidekova, Draga Toncheva, Anastas Pashov, Diyana Belejanska, Yavor Zhelev, Mariya Petrova, Shima Mehrabian, Latchezar Traykov

**Affiliations:** 1 Department of Medical Genetics, Medical Faculty, Medical University-Sofia, 1431 Sofia, Bulgaria; dimitase@gmail.com (D.S.); m.mihaylova@medfac.mu-sofia.bg (M.M.); svhadjidekova@medfac.mu-sofia.bg (S.H.); dragatoncheva@gmail.com (D.T.); 2 Department of Genetics, Faculty of Biology, Sofia University ‘St. Kliment Ohridski’, 1164 Sofia, Bulgaria; mikaela.stancheva@gmail.com; 3 Institute of Plant Physiology and Genetics, Bulgarian Academy of Sciences, 1113 Sofia, Bulgaria; antov8107@abv.bg; 4 Bulgarian Academy of Sciences, 1000 Sofia, Bulgaria; 5 Department of Immunology, Institute of Microbiology, Bulgarian Academy of Sciences, 1113 Sofia, Bulgaria; a_pashov@microbio.bas.bg; 6 Department of Neurology, University Hospital ‘Alexandrovska’, 1431 Sofia, Bulgaria; dianabelejanska@gmail.com (D.B.); yavor.zhe@gmail.com (Y.Z.); dr.mpetrova@yahoo.com (M.P.); shima_meh@yahoo.com (S.M.); traykov_l@yahoo.fr (L.T.)

**Keywords:** dementia, whole-exome sequencing, pathogenic variants, hyperammonemia, urea cycle disorders, argininosuccinate synthase 1 deficiency

## Abstract

The genetic bases of Alzheimer’s disease (AD) and frontotemporal dementia (FTD) have been comprehensively studied, which is not the case for atypical cases not classified into these diagnoses. In the present study, we aim to contribute to the molecular understanding of the development of non-AD and non-FTD dementia due to hyperammonemia caused by mutations in urea cycle genes. The analysis was performed by pooled whole-exome sequencing (WES) of 90 patients and by searching for rare pathogenic variants in autosomal genes for enzymes or transporters of the urea cycle pathway. The survey returned two rare pathogenic coding mutations leading to citrullinemia type I: rs148918985, p.Arg265Cys, C>T; and rs121908641, p.Gly390Arg, G>A in the argininosuccinate synthase 1 (*ASS1*) gene. The p.Arg265Cys variant leads to enzyme deficiency, whereas p.Gly390Arg renders the enzyme inactive. These variants found in simple or compound heterozygosity can lead to the late-onset form of citrullinemia type I, associated with high ammonia levels, which can lead to cerebral dysfunction and thus to the development of dementia. The presence of urea cycle disorder-causing mutations can be used for the early initiation of antihyperammonemia therapy in order to prevent the neurotoxic effects.

## 1. Introduction

Dementia refers to a clinical syndrome characterized by a progressive loss of cognition that interferes with the individual’s ability to function independently, which has negative effects on the patient, relatives and society. Diagnosing dementia is complex and it involves clinical evaluation, cognitive decline screening, laboratory assessment and structural imaging. Current treatment of the condition is not entirely effective and depends on the time of diagnosis. The situation becomes even more alarming with population aging, since it is expected that the number of dementia cases will triple by 2050. This requires novel approaches to prevent or delay the onset of the disease, which can be facilitated by performing genetic studies and implementing the obtained results.

Dementia presents with different subtypes with varying levels of heritability. The most common form of dementia is Alzheimer’s disease (AD), which is also one of the most common age-related diseases. It is defined as a slowly progressive neurodegenerative disease that mostly affects the medial temporal lobe and the neocortical structures. AD’s pathological determinants are senile plaques (related to the accumulation of amyloid-β peptides—Aβ), neurofibrillary tangles (of hyperphosphorylated microtubule associated tau protein) and the loss of neurons [1,2].

According to the age of onset, AD can be early-onset (diagnosed before the age of 65) and late-onset (diagnosed after the age of 65). Early-onset AD accounts for about 5% of all cases. It can be familial, usually autosomal-dominant, and caused by highly penetrant mutations in single genes. Approximately 10–15% of the disease-causing mutations in early-onset AD are found within the *APP* (amyloid precursor protein), *PSEN1* (presenilin 1) and *PSEN2* (presenilin 2) genes, the products of which regulate the production of Aβ [3]. Most of the remaining cases have mutations found in other genes (*GRN*, *MAPT*, *TREM2*, *SORL1*, *CLU*) or the causing mutation remains unknown [4,5,6]. Late-onset AD is a multifactorial condition with a strong genetic predisposition (with 40 to 80% heritability) [7]. The most powerful genetic risk factor for late-onset AD is the presence of the ε4 allele of the *APOE* (apolipoprotein E) gene. In addition to *APOE* ε4, whole genome studies of late-onset AD patients have determined that infection pathways, amyloid precursor protein processing and lipid metabolism, as well as endocytosis and tau protein processing, are mostly genetically associated with late-onset AD [8,9].

The second most common cause of early-onset dementia is frontotemporal dementia (FTD) [10]. It is a group of hereditary neurodegenerative disorders characterized by progressive changes in behavior, personality, language and motor function with involvement of the frontal and temporal lobes. Between 20 and 50% of FTD patients have a strong family history, often with an autosomal dominant type of inheritance. Mutations in the *MAPT*, *GRN* and *C9orf72* genes are found in 60% of familial FTD cases. Less than 5% of autosomal-dominant cases of FTD are due to mutations in *VCP*, *CHMP2B*, *TARDBP*, *FUS*, *SQSTM1*, *CHCHD10*, *TBK1*, *OPTN*, *CCNF* and *TIA1* [11,12,13].

The differential diagnosis of Alzheimer’s disease and frontotemporal dementia is often challenging. This is probably due to the clinical and pathological overlap of these disorders [14], such as the presence of hyperphosphorylated tau protein in approximately 40% of FTD cases [15]. AD and FTD phenotypic overlaps are probably mirroring the pleiotropic effect of the disease-related genes, as demonstrated for many neurodegenerative disorders [16]. In particular, it has been determined that FTD-causing genes, such as *MAPT* and *VCP*, are associated with AD risk, as the genetic crosslinks between these two forms of dementia spread far beyond the mentioned genes [17,18].

The genetic knowledge about Alzheimer’s disease and frontotemporal dementia is constantly increasing, which is not the case for atypical cases that do not fit these diagnoses. Thus, the genetics of non-AD and non-FTD dementia have been analyzed in a small number of cases and only for handful of genes. One of these studies includes 11 Finnish families with cases of unspecified dementia, who were tested for *APOE* genotypes as well as *C9orf72* expansions, the last being found in one of the unspecified dementia families [19]. Another study involving a patient with unspecified dementia was focused on coding mutations in dementia-related genes (*PSEN1*, *PSEN2*, *APP*, *MAPT*, *APOE*, *GRN*, *TARDBP*, *CHMP2B*, *TREM2*, *VCP*, *FUS*). The survey did not detect any known pathogenic mutation in the analyzed genes in the patient with unspecified dementia [20].

Another field that is underrepresented in dementia research is the study of high levels of ammonia as a potent neurotoxin [21], although increased blood ammonia levels have previously been established in patients with Alzheimer’s disease [22]. Ammonia is produced by the intermediary metabolism of amino acids or other nitrogen-containing compounds. It is considered that human adults produce about 1000 mmol of ammonia daily. Increased serum ammonia levels can cross the blood–brain barrier causing altered mental status or encephalopathy. Hyperammonemia is related with the reduced cerebral metabolic rate of glucose; disturbance of glutamatergic or GABAergic neurotransmission; and mitochondrial dysfunction; and dysregulated inflammation [23,24]. One of the hyperammonemia-causing factors is represented by urea cycle disorders, which are a group of inborn errors of ammonia detoxification/arginine synthesis [25]. They are due to recessive mutations in the genes for the catalytic enzymes carbamoylphosphate synthetase I (*CPS1*), ornithine transcarbamylase (*OTC*), argininosuccinic acid synthetase (*ASS1*), argininosuccinic acid lyase (*ASL*) and arginase (*ARG1*); one cofactor-producing enzyme, N-acetyl glutamate synthetase (*NAGS*); or two amino acid transporters, ornithine translocase (*ORNT1*) and citrin *SLC25A13* (aspartate/glutamate carrier; solute carrier family 25, member 13) [26]. Complete deficiency of these enzymes causes severe hyperammonemia in the neonatal period, whereas partial enzyme deficiencies may be associated with higher ammonia levels later in life [26]. The elevations of citrulline in argininosuccinate synthetase deficiency (citrullinemia type I), argininosuccinate in argininosuccinate lyase deficiency and arginine in arginase 1 deficiency are used as screening parameters and are included in newborn screening programs for the detection of these disorders [27]. Regarding the treatment of urea cycle disorders, there are different therapeutic approaches which aim to prevent the accumulation of ammonia, reduce the production of excessive nitrogen, redirect waste nitrogen to alternative metabolic pathways and replace arginine. Depending on the onset and severity, urea cycle disorders are treated by liver transplantation, hemodialysis, arginine, sodium benzoate, sodium phenylbutyrate and sodium phenylacetate, diet, etc. These therapies contribute to increased life expectancy and can improve the neurological consequences [28,29].

Taking into account the insufficiently disentangled genetic knowledge of non-AD, non-FTD dementia on one hand and the relative scarcity of the understanding of the neurological effects of hyperammonemia on the other, we have analyzed the presence of pathogenic variants in urea cycle disorder-genes in high-coverage pooled whole-exome sequencing (WES) data from 90 Bulgarian patients.

## 2. Materials and Methods

For the purpose of this study, blood was sampled from 90 patients (46 females and 44 males) with undefined (non-AD, non-FTD dementia) recruited at the Department of Neurology, University Hospital ‘Alexandrovska’, Sofia, Bulgaria. The study was conducted in accordance with the Declaration of Helsinki and approved by the Ethical Committee of Medical University of Sofia, Bulgaria (protocol N 1879/17.06.2021). Patients enrolled in the study underwent a careful clinical and neurological examination and a detailed history. They were administered a battery of validated neuropsychological tests, assessed for cognitive abilities and coping strategies, tested for cerebrospinal fluid markers (amyloid-β, total tau and phosphorylated tau) and examined by magnetic resonance imaging.

Patient inclusion criteria were signed informed consent, being over 40 years of age and having a Mini-Mental Status score (MMSE) ≥ 20. In addition, the selection of non-AD and non-FTD dementia was based on the current diagnostic criteria for Alzheimer’s disease NINCDS- ADRDA [30,31,32] and the consensus criteria for FTD [33,34]. Patients were excluded from the study based on the presence of one of the following criteria: normal cognitive functioning; depression; other disease that affects cognitive functions (epilepsy, head injuries, etc.); history of alcohol or other substance abuse in the past 2 years; primary sensory deficits; the use of experimental medication or other conditions that precluded the diagnosis of FTD.

The mean age at onset of the cohort was 62.2 ± 11.0, ranging from 40 to 84 years. The average baseline MMSE score was 24.7 ± 3.1.

DNA samples from the patients were isolated from blood by phenol–chloroform extraction. Equimolar amounts of each DNA sample were used for constructing a pooled DNA sample.

The whole-exome sequencing was performed by using Illumina^®^ SBS technology, and sequencing libraries were generated using Agilent ^®^ SureSelect Human All ExonV6 kit (Agilent Technologies, Santa Clara, CA, USA). The total number of sequenced raw reads was 163,914,926, from which low-quality reads (Qscore ≤ 5) and reads containing adapters were removed. Overall, 97.8% of the remaining reads had Phred values larger than Q20. The average read length was ∼150 bp and the achieved mean coverage was 250×, ensuring the detection of low-frequency alleles. The reads were aligned to the reference genome GRCh37/hg19. The .vcf files were annotated using wANNOVAR [35].

Pooled whole-exome sequencing does not give information about the individual exomes, but it can determine the frequency of the present genetic variants. Those of them which have strong pathogenic effects can explain the pathogenesis of dementia in part of the analyzed patients.

We have screened only the variants in urea cycle disorder-genes: *CPS1*, *ASS1*, *ASL*, *ARG1*, *NAGS*, *ORNT1* and *SLC25A13* (citrin). The *OTC* gene was not considered, since it is localized on the X-chromosome and the male:female ratio of the analyzed patients is not equal. Among these variants, we have considered only those which are reported as pathogenic in ClinVar. The pathogenicity of the variants was also evaluated by using the in silico pathogenicity prediction tool Combined Annotation Dependent Depletion (CADD) score [36]. The 3D structure models of wild-type and mutant proteins was predicted by using SWISS-MODEL [37].

The significance of the frequency differences of the urea cycle pathogenic variants in the analyzed pool and in control exomes was evaluated by the chi-squared test. As in other genetic surveys of age-related disorders, the present study raises the issue of appropriate controls for comparison. The best scenario in this case is the use of age-matched healthy subjects, which in most cases is not feasible. In the present study, we have used data from gnomAD, the most widely used publicly available collection of population variation—in particular, the data for 1335 Bulgarian exomes from gnomAD v.2.1.1. The database does not contain data about the age of all samples. The available information shows that the largest part of the samples in the entire gnomAD v.2 exome dataset are aged 50–60 years (24,626 out of 85,462 samples).

## 3. Results

The whole-exome sequencing of the pooled DNA sample of 90 patients with non-AD, non-FTD dementia revealed 453,631 variants, 424,692 of which are single nucleotide variants. The urea cycle genes (*CPS1*, *ASS1*, *ASL*, *ARG1*, *NAGS*, *ORNT1*, citrin *SLC25A13*) contain 139 variants. Two of the coding variants in autosomal urea cycle genes are reported as pathogenic in ClinVar: rs148918985, C>T, p.Arg265Cys; and rs121908641, G>A, p.Gly390Arg, both located in the argininosuccinate synthetase-1, *ASS1*, gene. The CADD scores (GRCh38.p14) of these variants are above 25, namely 26.6 for rs148918985, C>T, p.Arg265Cys and 29.6 for rs121908641, G>A, p.Gly390Arg, which shows that they are deleterious.

The established pathogenic urea cycle variants have less than a 0.001 MAF (minor allele frequency) in gnomAD and are not found in a homozygous state in the database. Among 85,462 global gnomAD exomes of individuals with known age, there are 59 heterozygote carriers of rs121908641, p.Gly390Arg aged from 30 to 80 years and only 1 heterozygote carrier of rs148918985, p.Arg265Cys in the 65–70 years range. The lack of individual homozygous for the mutations and the relatively high age of the heterozygote carriers presumes that the presence of these variants can be associated with age-related diseases, such as dementia.

The frequency of the variants in the analyzed pool and in gnomAD v2.1.1 Bulgarian control exomes, as well as the results of the chi-squared test, are summarized in Table 1. The variants show a statistically significant difference in frequency in the non-AD, non-FTD dementia pool and the gnomAD Bulgarian exomes, which potentiates their role in disease pathogenesis.

The *ASS1* gene encodes argininosuccinate synthetase-1, which is a cytoplasmic homotetramer expressed mainly in the cytosol of the periportal hepatocytes where the urea cycle takes place, as well as in the kidney and urinary bladder. It catalyzes the formation of argininosuccinate from citrulline and aspartate. The structure of the protein consists of three domains: a nucleotide-binding domain, a synthetase domain and a C-terminal helix involved in oligomerization [38].

**Figure 1:**
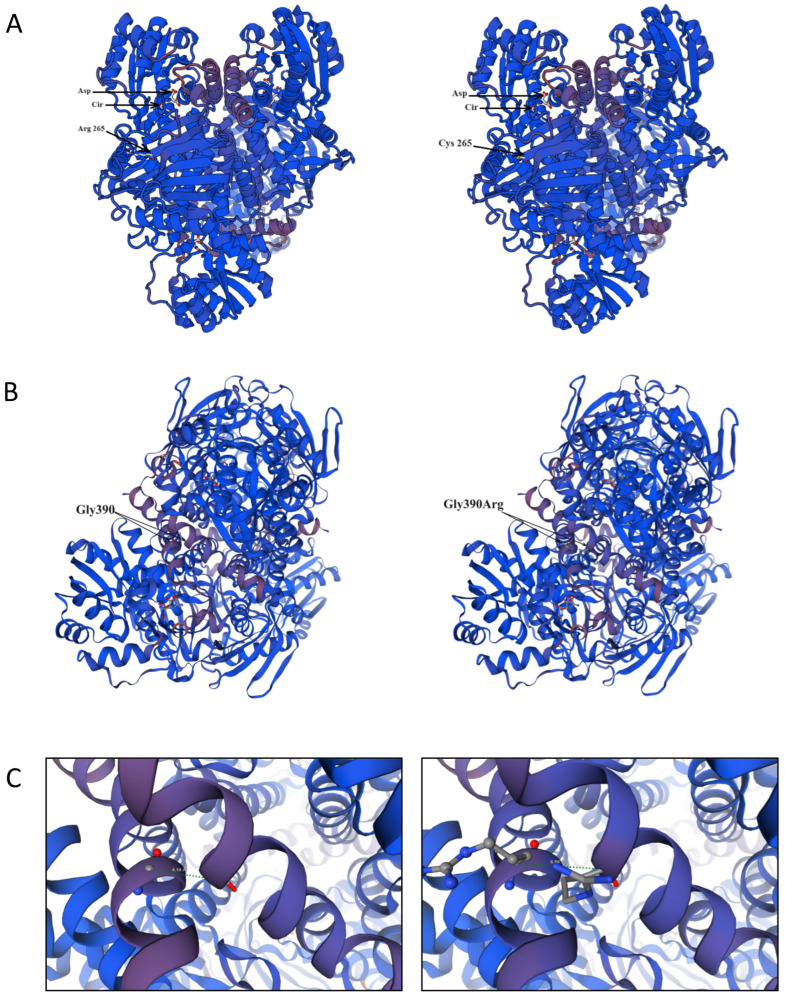
SWISS-MODEL structure of the ASS1 protein: (**A**) wild-type structure of ASS1 in complex with aspartic acid and citrulline (**left panel**) and mutant type Arg265Cys in complex with aspartic acid and citrulline (**right panel**), aspartic acid and citrulline are shown in sticks, Asp—aspartic acid, Cir—citrulline; (**B**) wild-type structure of ASS1 (**left panel**) and mutant-type Gly390Arg (**right panel**), aspartic acid and citrulline are shown in sticks; (**C**) close-up view of the distance (green dotted line) between two adjacent subunits in wild-type ASS1 (Gly390–Gly390)—4.14 Å (**left panel**), and in the mutant type (Arg390–Arg390)—5.80 Å, respectively (**right panel**).

The SWISS-MODEL predicted structures of the argininosuccinate synthetase-1 enzyme wild- and mutant-type Arg265Cys and Gly390Arg are represented in Figure 1. The Arg265Cys amino acid change (of the polar, basic, positively charged arginine with the polar, noncharged cysteine residue) is located in the 11th β sheet (from 265 to 271 amino acid position) (Figure 1A). Previous structure analysis has determined that Arg265 forms two hydrogen bonds with Trp210, as well as a cation–π interaction with Tyr207, which is further involved in extensive hydrogen bond network locking three crucial residues of the citrulline binding site (Ser189, Glu191 and Tyr282). Thus, we can consider that the Arg265Cys amino acid change indirectly affects the citrulline binding site of the argininosuccinate synthetase-1 enzyme [39]. The Gly390Arg amino acid change (of the nonpolar and neutral glycine with arginine) is in the last helix (385–406 amino acid position) of the C-terminal catalytic/multimerization domain of the protein (Figure 1B) and leads to an increased distance between two adjacent subunits (Figure 1C). By disturbing the secondary structure of the key oligomerization helix, the Gly390Arg substitution leads to enzyme inactivation.

## 4. Discussion

Dementia is a serious burden for patients themselves, relatives, professionals and the health system. Two major types of dementia, Alzheimer’s disease and frontotemporal dementia, have been the subject of many comprehensive genetic studies, aiming to facilitate risk estimation, early diagnosis and the introduction of new therapeutic measures. The dementia cases that do not fall within these diagnoses are not well studied genetically and the occurrence of pathogenic variants among them can reveal new insights into the genetic etiology and molecular mechanisms of dementia in general. Another aspect deserving special interest in the study of the multifaceted etiology of dementia is high levels of ammonia, which has demonstrated toxicity in CNS function. A congenital cause of hyperammonemia is urea cycle disorders, which can vary in onset and severity depending on the level of enzyme deficiency [40].

Motivated by this, we performed high-coverage WES of a pooled DNA sample of 90 Bulgarian dementia patients, who are not diagnosed with Alzheimer’s disease and frontotemporal dementia, and then we searched for pathogenic variants in urea cycle disorder genes. Among the obtained 424,692 single nucleotide variants, 139 were found within autosomal urea cycle genes (*CPS1*, *ASS1*, *ASL*, *ARG1*, *NAGS*, *ORNT1* and citrin *SLC25A13*). Of these, the variants rs148918985, C>T, p.Arg265Cys and rs121908641, G>A, p.Gly390Arg, both in the argininosuccinate synthetase-1 (*ASS1*) gene are reported as pathogenic in ClinVar and are deleterious according to the CADD score. Based on frequency, the variants are rare (gnomAD MAF < 0.001) and significantly different from the Bulgarian gnomAD v2.1.1 exome controls.

An enzymatic analysis of purified wild-type and mutant ASS by a bacterial in vitro expression study established that both mutated ASS-p.Arg265Cys and p.Gly390Arg lacked any significant enzymatic ASS activity, having below 2% of the wild-type protein enzymatic activity [41]. Later studies have established that rs148918985, C>T, p.Arg265Cys leads to a reduction in enzyme activity due to the indirect distortion of the extensive hydrogen bond network in the citrulline binding site [42], whereas rs121908641, G>A, p.Gly390Arg affects the oligomerization helix of the gene product and renders the enzyme inactive [39].

Regarding the phenotypic effect, mutations in the *ASS1* gene leading to enzyme deficiency determine the rare autosomal recessive disease citrulinemia type I. It is characterized with elevated blood citrulline levels 2000–5000 μmol/L (normal < 60), the accumulation of ammonia and glutamine and the increased excretion of orotic acid in the urine. The clinical phenotype varies based on the residual enzyme activity and can present in the following forms: neonatal acute (“classic”) form; milder late-onset (“non-classic”) form; a form that begins during or after pregnancy; and a form without symptoms or hyperammonemia. The symptoms in the neonatal form appear shortly after birth and include hyperammonemia, progressive lethargy, poor feeding, vomiting and signs of increased intracranial pressure. Neonatal citrullinemia type I can lead to life-threatening encephalopathy if not immediately treated. The milder late-onset patients exhibit delayed mental and physical development and chronic intermittent hyperammonemia during childhood and adulthood [26,43]. Individuals with residual enzyme activity can present with a biochemical phenotype but no clinical phenotype. In the expanded newborn screening, they show elevations of plasma citrulline below the range of the classical form of the disease and have consistently elevated plasma citrulline levels.

The citrullinemia type I variant rs148918985, C>T, p.Arg265Cys, *ASS1* was initially associated with the classical form of citrulinemia type I [41], but subsequent studies have suggested that the variant may allow some residual enzyme function [42]. The other *ASS1* variant—rs121908641, G>A is the most common globally distributed variant causing citrullinemia type I [41,44]. This variant has been associated with a severe and early-onset phenotype, as it has been observed in homozygous state in numerous cases in neonatal citrullinemia type I patients [45]. It is interesting to note that the identified *ASS1* variants were found in a compound heterozygote diagnosed with citrullinemia type I at 12 months of age and with clinical symptoms of the late-onset form of the disease [42]. The presence of a patient with such genotype within our sample can be confirmed only by the analysis of individual DNA samples. Still, the detected *ASS1* variants cause reduced argininosuccinate synthetase deficiency even in a heterozygous state, which then leads to increased ammonia levels exerting a toxic effect on the brain [46]. This can be supported by many recent reports that have identified symptomatic heterozygotes with mild and unspecific symptoms, occurring later in life in many disorders, including neurological diseases [47].

**Figure 2:**
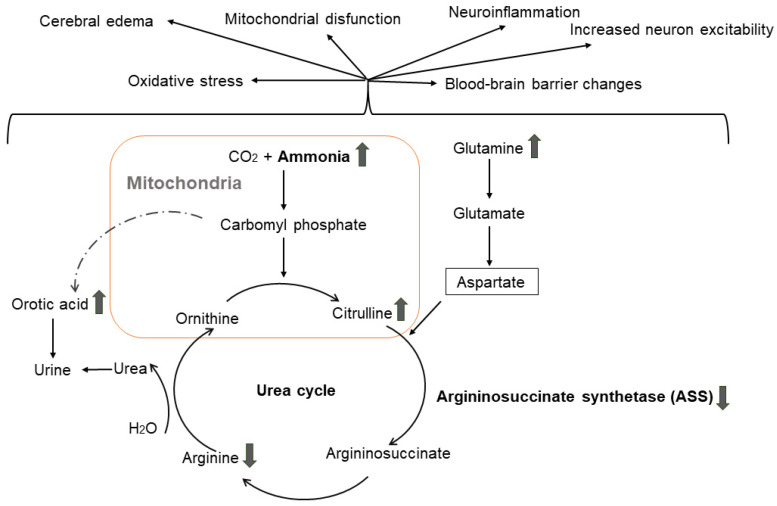
Argininosuccinate synthetase deficiency in the urea cycle, resulting in the accumulation of citrulline, ammonia, glutamine and orotic acid and effects on the central nervous system (upper arrows indicate increased level, lower arrows indicate decreased level/activity).

The proposed pathogenetic mechanism by which the established *ASS1* gene variants can cause dementia is represented in Figure 2. The presence of rs148918985, C>T, p.Arg265Cys and rs121908641, G>A, p.Gly390Arg results in residual or absent enzyme activity, respectively. The metabolic block leads to increased levels of ammonia, citrulline, glutamine and orotic acid [25] and decreased plasma levels of arginine. Hyperammonemia leads to depolarization of the resting membrane potential of neurons [48], changes in blood–brain barrier morphology [21], the elevation of pro-inflammatory cytokines, reduced antioxidant enzyme activity and inhibition of the mitochondrial electron transport chain (by inhibiting cytochrome c oxidase, complexes I–IV, superoxidase dismutase and glutathione peroxidase). Furthermore, the dysfunction of glutamate (precursor of glutamine and major excitatory neurotransmitter in the central nervous system) causes increased neuron excitability [49], and glutamine accumulation is associated to cerebral edema [50].

Depending on the time of exposure and dosage, the increased ammonia levels and concomitant metabolic consequences of the reduced argininosuccinate synthetase deficiency in the CNS lead to the development of neurological symptoms common to dementia such as memory deficits and cognitive decline. Based on these facts, the high levels of ammonia caused by mutations in urea cycle genes explain the pathogenesis of dementia in certain cases which cannot be diagnosed as AD or FTD.

## 5. Conclusions

Dementia is a debilitating neurological condition with cognitive decline, a heterogeneous nature and a complex etiology. Two insufficiently studied aspects in dementia research are genetic alterations in cases which do not fit within major dementia types such as Alzheimer’s disease and frontotemporal dementia, as well as the role of hyperammonemia in the development of dementia symptoms. Our pooled whole-exome sequencing data from 90 patients with non-AD, non-FTD dementia propose that rs148918985, C>T, p.Arg265Cys and rs121908641, G>A, p.Gly390Arg in the urea cycle *ASS1* gene play a role in the development of the disorder. These variants cause the autosomal recessive disorder citrullinemia type I and are not associated with the development of Alzheimer’s disease or frontotemporal dementia. The Arg265Cys and Gly390Arg variants lead to partial and complete enzyme inactivation, respectively. Thus, their presence in single or compound heterozygosity leads to high levels of ammonia that can act as a potent neurotoxin causing cerebral dysfunction. This possible metabolic pathogenetic mechanism for the development of dementia should be further investigated in individual samples and correlated with the levels of ammonia. In future, the presence of urea cycle mutations can be used for the early initiation of antihyperammonemia therapy in mutation carriers, in order to prevent the neurotoxic effects.

## Figures and Tables

**Table 1:** Pathogenic variants in autosomal urea cycle genes found in WES of pooled 90 patients with non-AD, non-FTD dementia.

Variant	Current Study			Bulgarian gnomAD Exomes, v.2.1.1			*p*-Value
	Allele Count	Allele Number	MAF (%)	Allele Count	Allele Number	MAF (%)	
rs148918985, *ASS1*, C>T, p.Arg265Cys	4	807	0.50	0	2666	0	0.002457
rs121908641, G>A, *ASS1*, p.Gly390Arg	13	1032	1.26	2	2568	0.08	0.000002218

## Data Availability

Data are contained within the article.
